# Thermoplasmonic‐Controlled Optical Filters Based on the Combination of Chiral Liquid Crystals and Metasurfaces

**DOI:** 10.1002/marc.202500339

**Published:** 2025-07-16

**Authors:** Federica Zaccagnini, Francesca Petronella, Michael E. McConney, Jonathan Slagle, Kwang‐Un Jeong, Timothy J. Bunning, Luciano De Sio

**Affiliations:** ^1^ Department of Medico‐Surgical Sciences and Biotechnologies Sapienza University of Rome Latina Italy; ^2^ National Research Council of Italy Institute of Crystallography CNR‐IC Strada Provinciale 35d Montelibretti, RM Italy; ^3^ Air Force Research Laboratory Materials and Manufacturing Directorate Wright‐Patterson Air Force Base Ohio USA; ^4^ Department of Polymer‐Nano Science and Technology Department of NanoConvergence Engineering Jeonbuk National University Jeonju Republic of Korea

**Keywords:** cholesteric liquid crystals, metasurfaces, plasmonics, tunable optical components

## Abstract

A novel class of photonic filters is obtained by integrating a large‐area optical plasmonic metasurface with a broadband cholesteric liquid crystal (CLC) Bragg reflector. By utilizing the photo‐thermal characteristics of the optical plasmonic metasurface, the reflection properties of the CLC layer can be manipulated. The thermally induced variation of the CLC's pitch and the refractive index produces a reversible blue‐redshift of the reflection band of about 80 nm. The synergistic interaction between these two effects enables the development of innovative optical color filters that integrate a light‐tunable mirror and a controllable optical absorber.

## Graphical abstract

A broadband cholesteric liquid crystal (CLC) combined with a bottom‐up metasurface absorber enables the realisation of thermoplasmonic‐controlled optical filters. These filters combine a CLC reflector for visible light with a metasurface absorber operating in the near‐infrared (NIR) range. These multi‐functional components are reconfigurable optical elements capable of simultaneously operating across two distinct regions of the electromagnetic spectrum: visible and NIR.

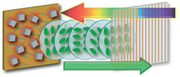

## 1 Introduction

Metasurfaces are electromagnetic wave modulators of planarly arranged subwavelength nanostructures on metallic or dielectric substrates. The features of these components can be affected by the selected materials, by tuning the height, width, and length of the meta‐atoms, or by varying their distribution or fill factor. The construction of these devices often relies on expensive top‐down techniques, such as lithography, that exhibit high sensitivity and guarantee a controlled deposition of the meta‐atoms. The low scalability of the devices that are limited to sizes from µm [1] to mm [2] hinders broad applicability. Growing interest is in developing a new generation of metasurfaces composed of randomly organized meta‐atoms using bottom‐up approaches, such as Langmuir–Blodgett techniques and self‐assembled monolayers [3]. However, the meta‐atom arrangements obtained through these bottom‐up techniques exhibit some drawbacks, including limited chemical temporal stability, a lack of physical robustness of the films under ambient conditions caused by the absence of strong molecular interactions, limited loading of organic compounds in the monolayer films for possible bioactivation purpose, and the required presence of specific compounds to allow the formation of arrays. Other surface engineering methods, such as polymer grafting or chemical vapor deposition, are also used to improve surface characteristics of the meta‐atoms distribution. However, the high costs of the instrumentation and production process, as well as the limited number or variety of materials that can be assembled, constitute crucial disadvantages of these commonly used bottom‐up techniques.

As an alternative, the Layer‐by‐Layer (LbL) technique has provided versatility, simplicity, and cost‐effectiveness assembly methodology since being introduced in the 1990s [4]. The LbL technique relies on multivalent molecules' repetitive, sequential adsorption on a surface via electrostatic or non‐electrostatic interactions to realize multilayer assemblies. Among existing deposition methods (dip‐coating, spin‐coating, spray‐coating, and perfusion), the immersive (dip‐coating) technique is the most widely used due to its ease and applicability to substrates with complex geometries. Immersing charged surfaces into oppositely charged solutions with intermediate washing steps provides well‐controlled structures and thicknesses of a bilayer film. Repeating this process multiple times allows one to build films of defined total thickness. This simple and efficient fabrication process is performed entirely in aqueous solutions, and the multilayer stability and properties can be controlled by varying the temperature, pH, and salt concentration of the polyelectrolyte (PE) solutions. The LbL technique meets the high demand for scalable and cost‐effective randomly organized metasurfaces. However, metasurfaces, constituted by a random or ordered arrangement of meta‐atoms (such as colloidal nanoparticles, NPs) on metallic or dielectric substrates [5], are passive, implying that these devices have fixed properties once fabricated. This is a significant limitation when integrating them in active photonic applications.

Two strategies have been conceived to overcome the passive nature of metasurfaces, including the use of external stimuli to modify structural aspects, such as varying the lattice constants, meta‐atom shapes, or spatial configurations, and the introduction of active materials as a surrounding medium. Metasurfaces combined with active materials like liquid crystals (LCs) offer a promising alternative. While they have a narrower tuning range, they are often preferred over dye‐based strategies due to their faster response times [6]. Indeed, an alternative approach to realize active metasurface‐based color filtering relies on the absorption anisotropy of dichroic dyes, which are produced through complex synthetic processes. In dye‐doped LC films [7], the orientation of dichroic dyes can be controlled using polarized light, inducing the reorientation of LC molecules [8, 9]. However, this method has notable limitations, including reduced transmission due to dye absorbance in the visible spectrum and the inability to achieve colorless and multicolor states. Conversely, the optical tunability of the metasurfaces combined with LCs in the visible range enables the development of fast‐tunable color filters.

LCs are soft and stimuli‐responsive molecules commonly used as an active material [6], exploiting their birefringence, responsiveness, and high transparency in the visible spectrum for dynamic photonic applications [10] including smart windows [11], color filters [12], colorimetric sensing [13], and smart devices [14]. Nematic LCs (NLCs) have been widely investigated for their birefringence as the refractive index associated with the long axis of the molecules differs from the short axis. Chiral NLCs (CNLCs) are 1‐d photonic crystals with periodic twists perpendicular to the substrate plane (Grandjean‐Cano configuration) [15], often used to develop films with vibrant coloration [16]. CNLCs, better known as cholesteric LCs (CLCs), are realized by adding chiral agents to NLCs, and their structure produces a circular polarization‐dependent reflective band (Bragg reflective band). CLC films reflect light with a polarization that has the same handedness of its twist and transmit the opposite handedness. The properties of the bandgap are easily tunable by controlling both the helical pitch and the refractive index of the CLCs [17] using thermal, electric, and photonic stimuli [18, 19].

The modulated optical properties of LC‐based metasurfaces have produced nanostructured transmission‐based, reflection‐based, and diffraction‐based color filters and colorful gamut mappings in video display construction [20, 21]. For example, De Sio et al. combined an array of gold nanospheres distributed on a glass substrate and a CLC film to realize a light‐controllable optical mirror [22]. Furthermore, we have recently reported and investigated the 46 nm photo‐thermal tunability of an innovative, large‐area, bottom‐up fabricated plasmonic metasurface‐based optical absorber made of silver nanocubes (AgNCs) randomly organized on a gold film, spaced through a dielectric layer, and overlaid with a planarly aligned NLC film [23]. In this study, we demonstrated that the artificial absorption peak of the metasurface, centered in the near‐infrared (NIR) region (700–800 nm), arises from the coupling between the localized surface plasmon resonance (LSPR) of AgNCs and the surface plasmon resonance (SPR) of the gold layer, mediated by the intermediate PE multilayer, PEM. Numerical simulations of the system, conducted using COMSOL Multiphysics 5.6, corroborated the experimental results and confirmed the trend of SPR peak modulation in response to the induced refractive index variation.

This work explores the potential of dynamic reflection‐based CLC color filters [24] enabled via thermoplasmonic heating. An innovative color filter integrating a random metasurface‐based light absorber and a CLC Bragg mirror is presented, operating in two distinct regions of the electromagnetic spectrum (NIR and visible). This multi‐optical component exhibits a reversible blue‐to‐redshift of approximately 80 nm of the CLC reflection band, induced by the thermoplasmonic effect generated by a NIR‐irradiation of the plasmonic metasurface. The observed color gamut variation demonstrates the ability to tune the green hue of CLC molecules in a non‐contact manner through thermal effects under unpolarized light. The system achieves significantly reduced response times compared to conventional thermochromic materials, which typically require several minutes to hours [25]. It is suitable for diverse applications, including non‐contact control in adaptive color control [26], responsive sensors, and actuators [16].

## 2 Results and Discussion

### 2.1 Layer‐By‐Layer Immersive Method for the Fabrication of Random Optical Metasurfaces

**FIGURE 1:**
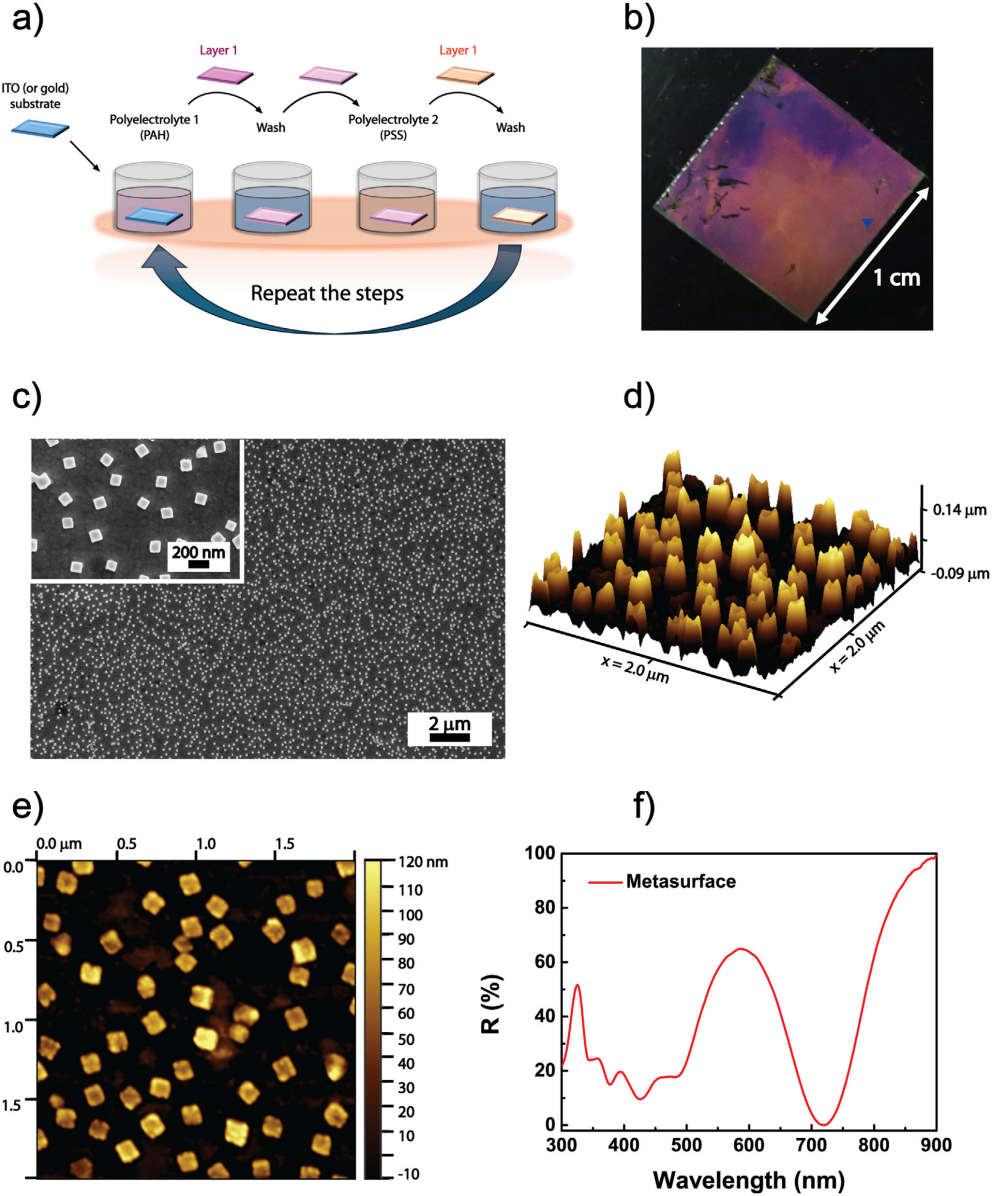
Fabrication and characterization of a random metasurface optical absorber exploiting a bottom‐up technique. (a) Schematic of the immersive and electrostatic LbL preparation method. (b) Photo of the fabricated metasurface. (c) Morphological characterization of the metasurface using scanning electron microscopy (SEM). (d) 3D and (e) 2D atomic force microscopy (AFM) analysis of the metasurface sample. (f) Reflection spectrum of the metasurface.

The first step in fabricating the thermoplasmonic‐controlled color filters is creating the metasurface optical component with optimal morphological and optical properties. The LbL assembly method (Figure 1a) is a bottom‐up technique that enables the realized nanostructures' scalability and reproducibility. This facile and safe technique was utilized to construct plasmonic NPs functionalized substrates with high sensitivity and fill fraction [23, 27, 28] A PEM was assembled using the LbL technique by immersing a glass/gold substrate in PE solutions, alternatively charged containing poly(allylamine hydrochloride), PAH (+) and poly(sodium styrene sulfonate), PSS (‐). The complete functionalization occurs after their final overnight immersion in the plasmonic NPs colloidal solution.

The protocol in ref. [23] was improved by immersing the PEM substrate overnight in the colloidal dispersion of AgNCs as the final step. Figure 1b shows the realized metasurfaces' homogeneous and bright pinkish color over a relatively large surface area (1 cm × 1 cm) compared to the usual mm dimensions of top‐down generated metasurfaces. The morphological characterization of the metasurface was performed using AFM and SEM analysis. The SEM micrographs in Figure 1c show an even and dense distribution of AgNCs (zoom in the inset) on the gold layer with an interparticle distance of 412.0 nm ± 63 nm and a size of 105 nm ± 12 nm. The high homogeneity and the calculated fill fraction of 11.7 ± 0.4 (calculated considering a region of interest of 312 µm^2^) greatly exceed previous results in ref [23]. AFM analysis in Figures 1d,e shows an even distribution of AgNCs with a height profile of 100 nm, consistent with the starting material.

The nanometer‐controlled LbL assembly of PEM is exploited to achieve the deposition and the self‐assembly of AgNCs. The random organization of well‐dispersed and highly dense AgNCs produces the realized bottom‐up metasurfaces' unique optical and thermo‐optical properties. Indeed, the plasmonic surface lattice resonances (SLR), given by the coupling of the SPR of the gold layer with the LSPR of the AgNCs array, produce the optical features of this device. The reflection spectrum (Figure 1f) highlights the presence of the AgNCs absorption peak at 485 nm and the stronger SLR peak (λ_M_) at 719 nm. The absorption efficiency, calculated as the intensity difference between the maximum reflected light and the reflection at 719 nm, is about 65%.

### 2.2 Thermal Response of the CLC

For a planarly aligned CLC film, the component of incident light matching the handedness of the molecular ordering in the CLCs is reflected, while the opposite‐handed component is transmitted [29]. This selective reflection of light is described by Bragg's law, which indicates that the mean wavelength of the reflection band (Bragg wavelength, λ_B_) depends on the pitch (*p*) of the helical structure and the effective refractive index (*n̅*) of the CLC.

(1) $$\lambda_{B}=\bar{n}\;*p$$

A CLC having a reflection band in the visible range (λ_B_ ∼ 500 nm) was selected for this preliminary study. The high birefringence 1825 CLC material exhibits a Δn = 0.42 at 598 nm [30], and a bandwidth (Δλ) of about 120 nm. It was selected for its high transition temperature of 120°C, which enabled a broader temperature working range.

**FIGURE 2:**
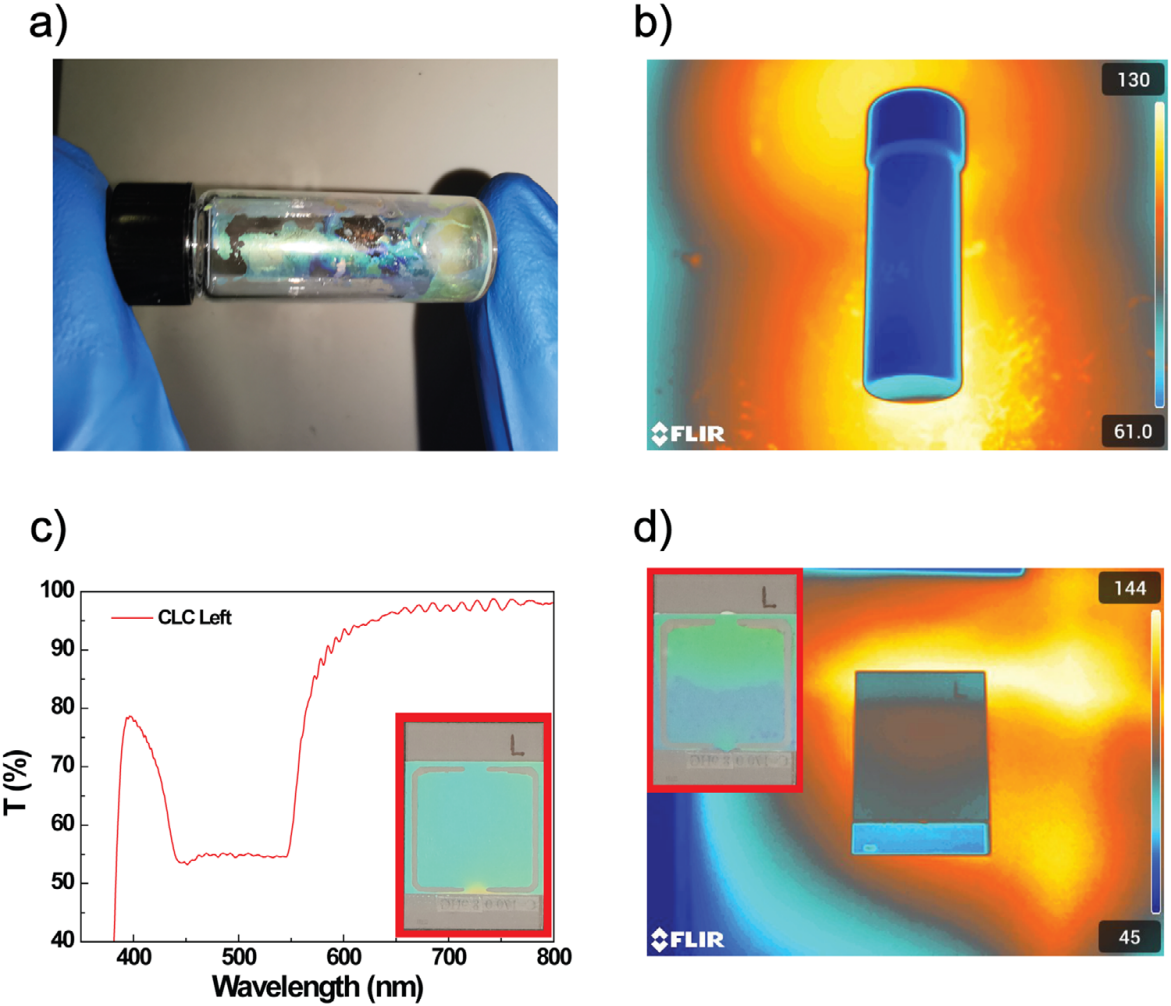
Hot plate heating of the CLCs. (a) Photograph of transparent vials containing a Left‐handed CLC heated by a hot plate at 115°C. (b) Thermographic image of the same vial on the hot plate. (c) Transmittance spectrum of the Left‐handed CLC cell, with a photo of the cell in the inset. (d) Thermographic image of a Left‐handed CLC cell on the hot plate at temperatures higher than 100°C, showing a chromatic change (inset photo).

Figure 2a shows a photo of a transparent vial containing a left‐handed CLC solution heated to a high temperature of 115°C on a hot plate. The distinctive colors of the CLC solution indicate that the chiral structures remain stable, confirming the high CLC‐iso transition temperature [31], which is slightly reduced by adding chiral dopants [32]. A thermographic image (Figure 2b) was captured to verify the temperature distribution, confirming that the lower part of the vial, which is in contact with the hot plate, reached the set temperature.

Figure 2c displays the room‐temperature transmission spectrum of a 10 µm thick planarly aligned glass cell infiltrated with the CLC, showing a Bragg reflection peak of approximately 50% centered near 500 nm. In the inset at the bottom right of Figure 2c, a photograph of the CLC cell shows the bright green color of the reflectance band. The thermal responsivity of the CLC sample was investigated by monitoring its optical response as the temperature was gradually increased using a hot plate (Figure 2d). A gradual chromatic shift (Figure S1) was observed as the temperature rose to 120°C, first blue‐shifting from the original green color to a bluish hue (inset of Figure 2d), followed by red‐tuning.

**FIGURE 3:**
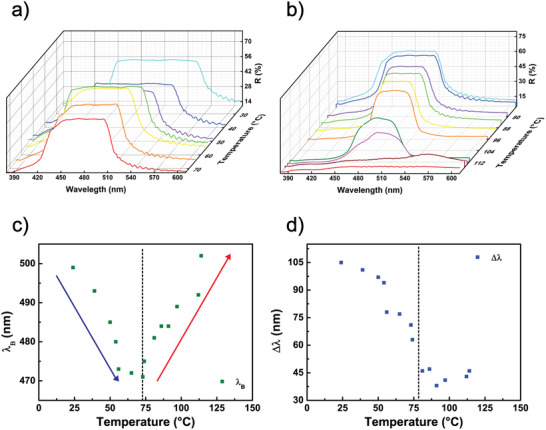
Thermal response of the CLCs. (a,b) Reflectance spectra of the CLC cell at various temperatures, showing a blueshift in the reflection band between 24°C and 73°C (a) and a redshift from 73 to 120°C (b). (c) λ_B_ position and (d) reflection bandwidth of the Left‐handed CLC cell as functions of temperature.

The reflection spectra of the sample under unpolarized light during heating are shown in Figure 3a,b. In the temperature range from 24°C to 73°C (Figure 3a), the λ_B_ undergoes a blueshift of 28 nm, a narrowing of the bandwidth by 34 nm, and a slight reduction of the intensity. Further increasing the temperature from 73°C to 120°C (see Figure 3b) leads to a redshift in λ_B_ of 31 nm and an additional narrowing of the reflective band of 25 nm, in parallel with a significant reduction of the band reflectance approaching the transition from chiral to isotropic phase. The system demonstrated excellent reversibility, returning to its original state when cooled to room temperature (see Video S1).

The data collected in these two regimes are summarized in Figure 3c,d, which display the λ_B_ position and reflective bandwidth as functions of temperature for the CLC sample, respectively. A dotted line highlights the temperature at which there is a transition from blue‐ to red‐shifting of the peak wavelength. This behavior can be explained by calculating the differential of the λ_B_ expression (see Equation (1)) as follows:

(2) $$\Delta \lambda_{B}=\Delta \bar{n}*p+\bar{n}*\Delta p$$

Two separate contributions can lead to changes in the wavelength, including changes in the average refractive index ($\Delta \bar{n}$) and to the helical pitch (Δp) of the CLCs. In the lower temperature range, the contribution from $\Delta \bar{n}$ dominates, leading to a blueshift as a direct result of the decreasing average refractive index. Conversely, in the higher temperature range, the contribution from Δp becomes more significant as above 73°C the helix of the CLC begins to unwind, causing an increase in pitch. As a result, $\Delta \lambda_{B}$ increases as well. Meanwhile, the reflection bandwidth decreases due to the ongoing reduction in the average refractive index, an effect that becomes more pronounced at higher temperatures. The bi‐directional thermal behavior of the CLC is quite intriguing and allows for the creation of innovative color filters with bidirectional control. This work focuses on inducing and controlling color changes by utilizing the thermoplasmonic properties of the optical metasurface.

### 2.3 Metasurface‐CLC Cell Construction

**FIGURE 4:**
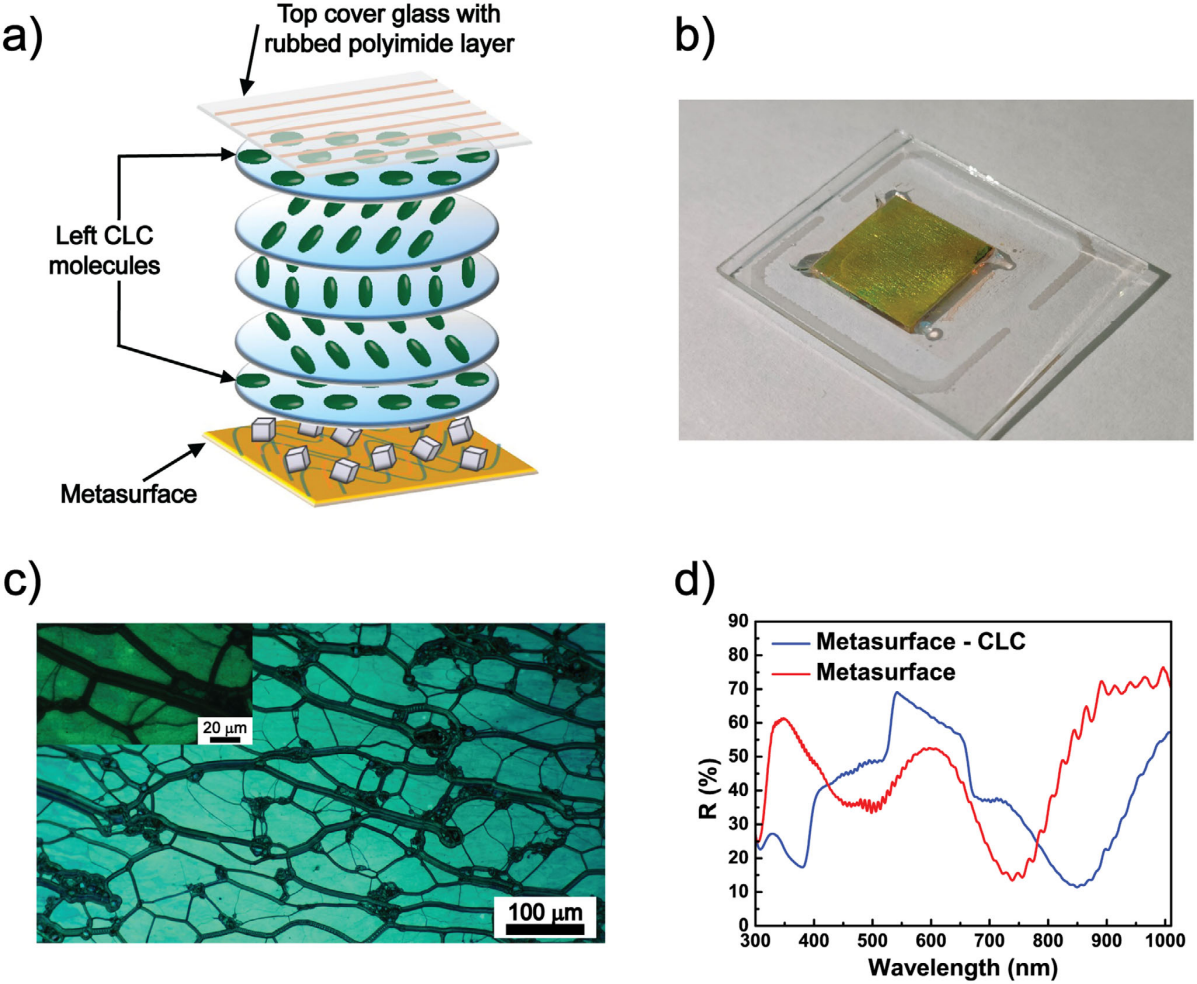
Fabrication of the metasurface‐CLC optical filter. (a) Schematic of the metasurface‐CLC system. (b) Representative photograph and (c) polarized optical microscopy images of the metasurface‐CLC sample at different magnifications. (d) Reflectance spectra of the empty metasurface cell (red curve) and after infiltration with the CLC (blue curve).

The photo‐thermal heating capability of the metasurface (see Section S2) was exploited to actively control the reflective properties of the CLC material, thereby creating a light‐controllable color filter. The high‐birefringence, high‐transition‐temperature CLC characterized in **Section** **2.2** was combined with the metasurface to form a metasurface‐CLC assembly (Figure 4a). This was achieved using a commercially available planarly aligned top‐cover glass (aligned with a rubbed polyimide layer), bonded to the metasurface using 10 µm glass spacers dispersed in NOA‐61 monomer. The components were secured through a UV‐initiated photopolymerization process. Figure 4b highlights that the color changes from the initial pink tone of the as‐fabricated metasurface (Figure 1b) to a yellow hue once the CLC is infiltrated into the cell. This noticeable shift is attributed to the large refractive index change in the medium surrounding the metasurface (from air, *n* = 1, to CLC, $\bar{n}$= 1.7) and the selective reflective properties of the CLC molecules [33]. The morphological analysis conducted using reflective polarized optical microscopy (POM) (Figure 4c) reveals the characteristic oily streaks of the planar texture of the CLCs. Due to these intrinsic structural defects of the CLCs, it is challenging to directly observe the alignment conditions of CLC molecules in close proximity to the AgNCs. Therefore, we refer to our previous work [23] where a similar optical metasurface was combined with NLCs. In this study, POM confirmed that a uniform planar alignment of the NLC was successfully achieved. Specifically, when the molecular director was aligned at 45° to the polarizer axis, the NLC‐metasurface system appeared as a uniform bright layer, indicating homogenous molecular orientation. Upon rotating the sample such that the director was aligned parallel (0°) to the polarizer, the observed dark field was punctuated by bright spots whose sizes were comparable to those of the AgNCs. This suggests localized disruptions in alignment. Furthermore, Mueller matrix polarimetry supported these observations by confirming that the misaligned regions correspond to the presence of AgNCs. This is attributed to the local deformation of the LC director field caused by the nanoscale geometry of the AgNCs, which induces anisotropic interactions with the surrounding NLC molecules.

The optical metasurface exhibits high sensitivity to changes in the refractive index of its surrounding medium [34]. Notably, even before CLC infiltration, the reflective spectrum of the empty metasurface cell (Figure 4d, red curve) is already red‐shifted by approximately 25 nm from 719 nm of the bare metasurface (Figure 1f) to 744 nm of the metasurface cell (Figure 4d, red curve)—and attenuated by about 10%. This behavior is attributed to two effects of the top‐cover glass substrate, which reduces the PEM gap between the AgNCs and the underlying Au layer due to its own weight and a different refractive index between the optical metasurface and the surrounding medium (with glass rather than air). Both of these effects are consistent with previous results [35]. After infiltrating the cell with CLC (Figure 4d, blue curve), the change in refractive index further shifts λ_M_ to 853 nm while the characteristic Bragg reflective band of the CLC appears in the visible range, centered around 600 nm. To prevent the overlap between the CLC reflection band and the AgNCs absorption peak at 485 nm, we used a slightly modified CLC material with a reflection band centered in the visible range (λ_B_ ∼ 600 nm), achieving clear spectral separation from the metasurface band centered at 719 nm and the absorption of AgNCs at 485 nm.

### 2.4 Photo‐Thermal Characterization of the Metasurface‐CLC Sample

**FIGURE 5:**
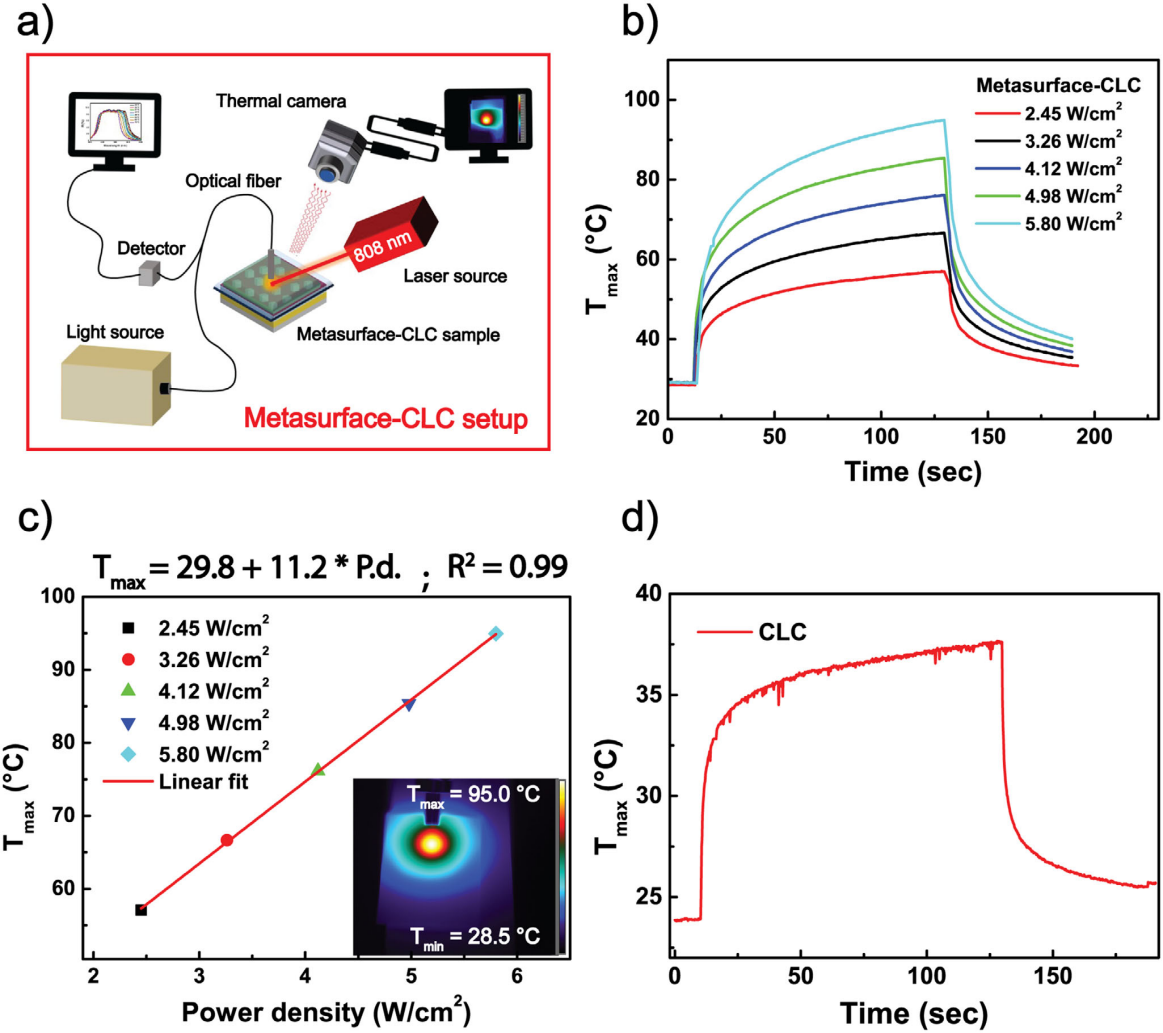
Photo‐thermal characterization of the metasurface‐CLC device. (a) Schematic of the experimental photo‐thermal setup. (b) Temperature increase observed upon irradiation of the optical metasurface with an 808 nm laser at varying power densities. (c) Maximum temperatures reached by the optical metasurface as a function of the experimental data points' NIR power density and linear fit (red line). Thermal image captured at the highest achieved temperature in the inset. (d) A temperature increase observed upon irradiation of the CLC cell with an 808 nm laser at a power density value of 5.80 W cm⁻^2^.

Figure 5a illustrates the experimental thermo‐optical setup used to induce the gradual photo‐thermal phase transition (from CLC to isotropic) of the metasurface‐CLC device, monitored through changes to the Bragg reflection band of the CLC film. The metasurface‐CLC sample was irradiated by a 808 nm laser. After adding the CLC to the metasurface sample, the absorption band of the metasurface is red‐shifted to a longer wavelength, thus justifying the selected wavelength of the 808 nm laser excitation. The thermo‐optical setup (Figure 5a) enables simultaneous monitoring of temperature distribution using a high‐resolution thermal camera and collection of the specular reflection spectrum of the system under investigation through a reflective spectrophotometer. The photo‐thermal response of the metasurface‐CLC device was monitored while irradiating for 2 min with the NIR laser at different power density values. Maximum surface temperatures ranging from 57.08°C to 95.0°C were recorded for power densities between 2.45 and 5.80 W cm⁻^2^ after the 2 min of NIR irradiation (see Figure 5b). The increase in temperature is approximately linear with power density, as highlighted in Figure 5c. The thermographic image (inset of Figure 5c) highlights the peak temperature of 95.0°C at 5.80 W cm⁻^2^. The comparison with the bare metasurface's photo‐thermal response in Figure S2, highlights that the metasurface‐CLC sample achieves higher temperature values at lower power densities. This behavior is justified by the 109 nm redshift of the metasurface peak once the CLC molecules are in close contact with the metasurface (Section 2.3). Remarkably, the photo‐thermal heating generated by the CLC‐metasurface, due to its unique perfect absorption behavior, allows for achieving higher temperatures compared to recently reported metasurfaces, as shown and discussed in the comparative Table SI1. To validate the photo‐thermal efficiency of the metasurface‐CLC device, control experiments were performed by irradiating for 2 min the bare CLC cell under an NIR laser at 5.80 W cm⁻^2^ power density value, used to control the Bragg band tuning; once the CLC molecules are infiltrated in the metasurface cell. The temperature increase demonstrates that the achieved temperature of 37.6°C is insufficient to modify the optical features of the CLC molecules, highlighting the crucial contribution of the metasurface thermoplasmonic effect (Figure 5d).

### 2.5 Thermoplasmonic Controlled Metasurface‐CLCs Component for Light Filtering Applications

The experimental setup shown in Figure 5a was used for the thermoplasmonic tuning of the metasurface‐CLC specimen under irradiation from a NIR laser source. 20 min of NIR laser irradiation at 5.80 W cm⁻^2^ were necessary to monitor the whole CLC‐to‐isotropic phase transition: higher power densities led to a sudden distortion of the optical properties. In comparison, lower power densities did not achieve the total phase transition. The high‐resolution thermal camera recorded the maximum temperature values on the metasurface‐CLC device.

**FIGURE 6:**
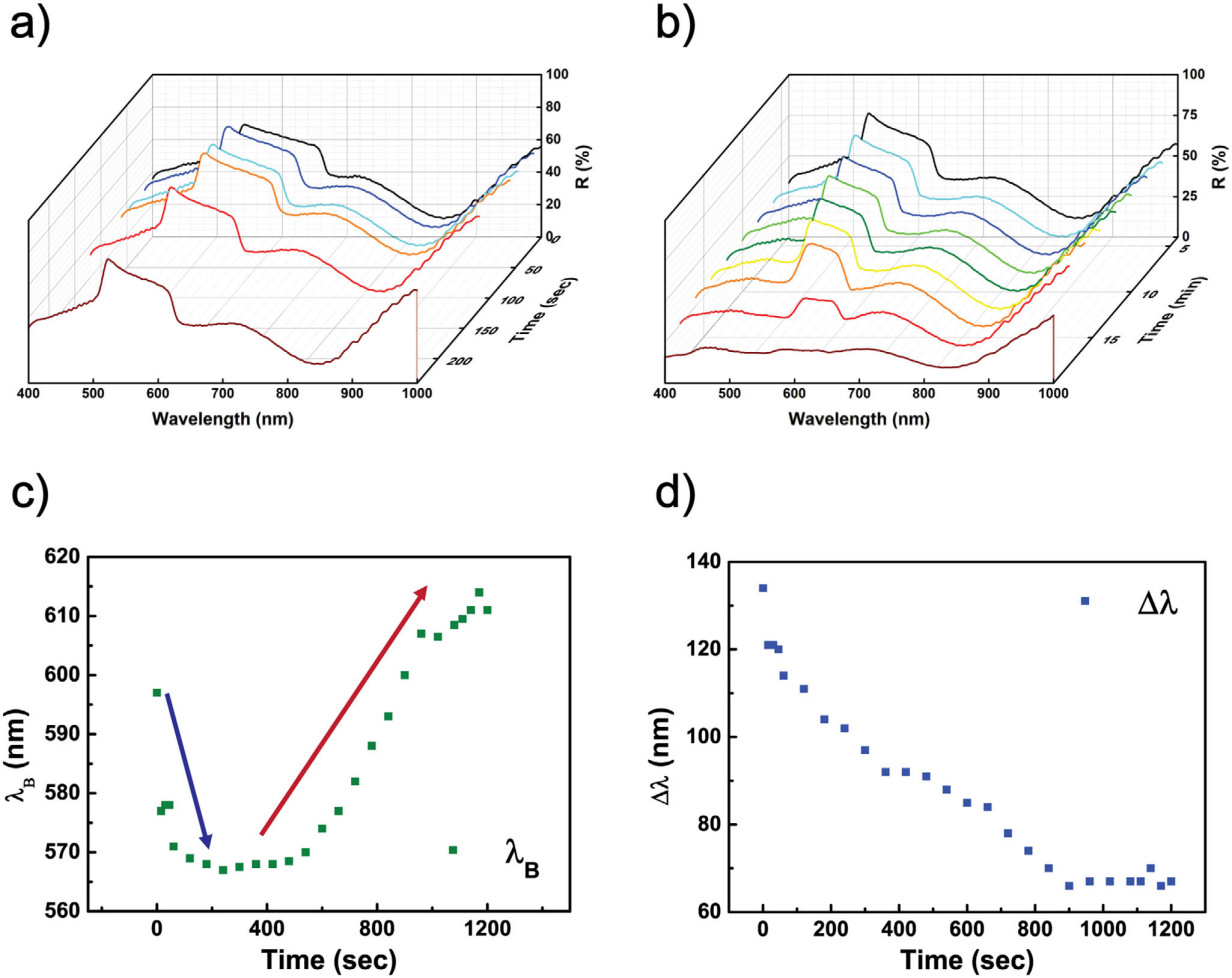
Thermoplasmonic‐controlled metasurface‐CLC optical filter: tunable CLC reflector. (a) Reflectance spectrum showing blueshift, and (b) redshift of the metasurface‐CLC sample under NIR laser irradiation at different temporal intervals. (c) Temporal variation of the CLC λ_B_ position and (d) bandwidth (Δλ) with temperature.

**FIGURE 7:**
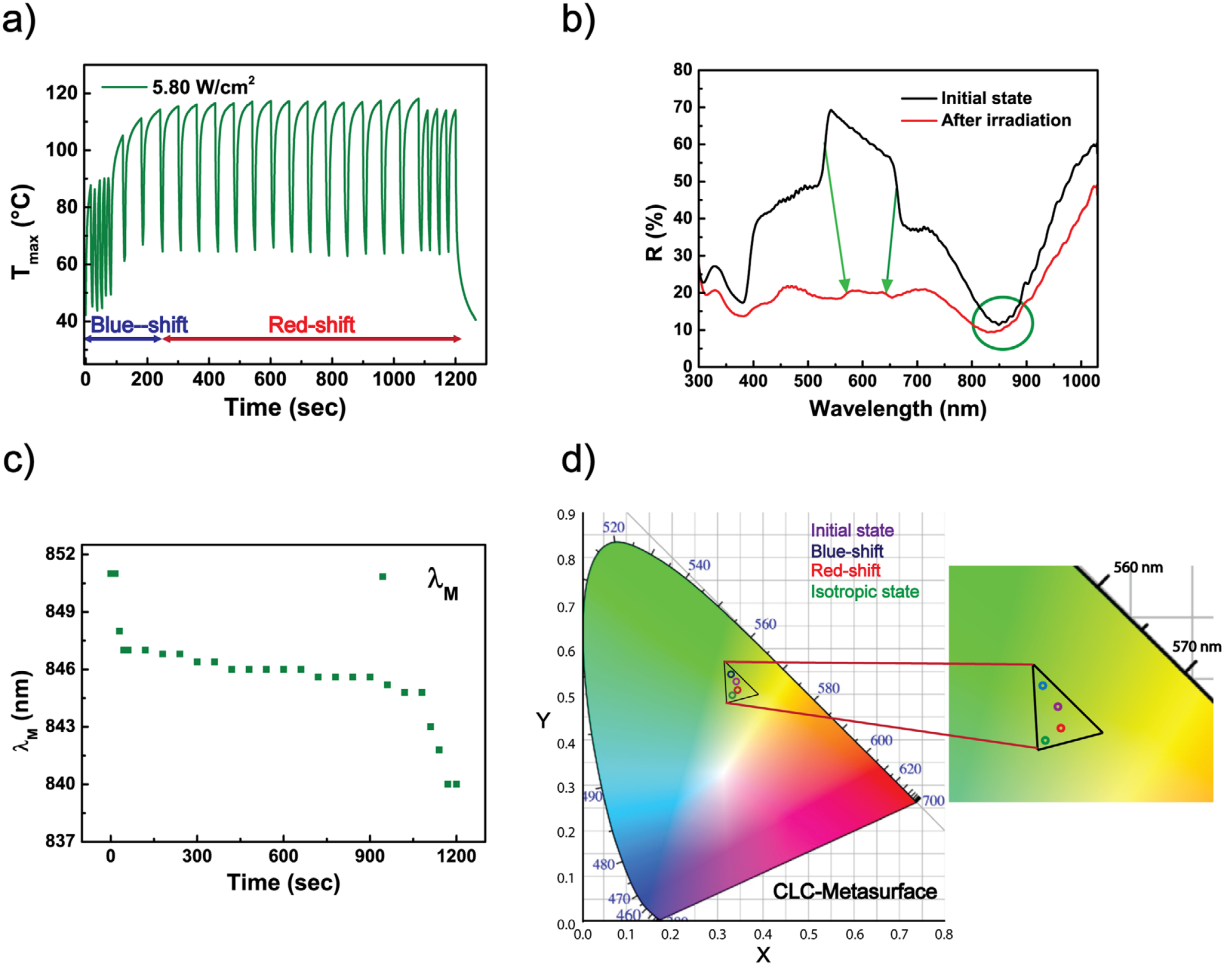
Thermoplasmonic‐controlled metasurface‐CLC optical filter: tunable metasurface absorber. (a) Photo‐thermal response of the metasurface‐CLC sample under 20 min of NIR irradiation at 5.80 W/cm^2^, with brief interruptions of the laser source for a few seconds each minute. (b) Reflectance spectra of the metasurface‐CLC cell before (black curve) and after (red curve) NIR laser irradiation. (c) Temporal variation of the center wavelength (λ_M_) in the absorption band of a metasurface optical absorber optical component with temperature. (d) Gamut color change on the CIE Chromaticity Diagram of the metasurface‐CLC color filter, along with a zoomed view of the CLC working range.

As a result of the gradual temperature increase during laser illumination, the CLC Bragg reflection experienced a blueshift of approximately 30 nm during the first 4 min (Figure 6a), at which point the maximum temperature was 114.2°C (see details in Figure 7a) This shift is attributed to a predominant decrease in $\Delta \bar{n}$ (as previously discussed, see Equation (2)).

After 4 min of laser illumination, the elongation effect, driven by the continuous increase in temperature to 118.2°C (see details in Figure 7a), began to outweigh the decrease, resulting in a redshift of 47 nm (Figure 6b), achieved after 20 min of continuous illumination. The blue‐redshift of the reflection band is better highlighted in Figure 6c, evidencing an overall shift (back and forth) of about 80 nm. The reflectance spectra exhibited high repeatability, with a standard deviation of less than 4%, confirming the robustness and stability of our measurements. The photo‐thermal‐induced reduction in the average refractive index also produces the gradual narrowing of the reflection bandwidth, as shown in Figure 6d. The as‐prepared broadband CLC exhibits a CLC‐isotropic phase transition temperature of approximately 120 °C (Figure 3b). When AgNCs are in close contact with the CLC layer, the CLC‐isotropic phase transition temperature is reduced to around 118 °C (see details in Figure 7a), likely due to local deformation of the CLC director field caused by the nanoscale geometry of the AgNCs. Figures 5, 6 demonstrate that photo‐induced heating on the surface of the metasurface substrate effectively propagates through the CLC volume, leading to measurable changes in the CLC's optical properties.

To collect the reflective spectra during laser illumination, the laser had to be temporarily turned off at each acquisition interval to prevent interference with the spectrophotometer measurements. However, as confirmed by the thermographic study (Figure 7a), these brief interruptions did not affect the behavior of the CLC. The maximum temperatures recorded varied at different intervals during the NIR irradiation, with notable fluctuations observed as the process progressed. After 18 min of NIR laser irradiation, a peak temperature of 118.2°C was reached before decreasing slightly during the final moments of irradiation, likely due to changes in the CLC's thermal conductivity as it approached the isotropic phase.

Figure 7b clearly illustrates the comparison between the reflectance of the device when the metasurface‐CLC is in its initial state (black curve) and at the end of irradiation (red curve) when the CLC reaches the isotropic phase. Additionally, the green circle in Figure 7b highlights the pronounced blueshift of the highly sensitive metasurface peak [34]. The photo‐thermal‐induced reduction in the average refractive index also produces a blueshift of λ_M_ by approximately 10 nm (Figure 7c). The photo‐thermal conversion capability of the metasurface‐CLC device was validated through the Roper model [36], considering the time constant value, τ, since it is inversely proportional to the photo‐thermal efficiency. The calculated value of 7.2 s highly exceeds the performance of the previous device discussed in ref [23], composed of the bottom‐up metasurface cell filled with E7 NLCs, due to the improved quality of the bottom‐up metasurface and to the unique features of the CLC molecules.

The performance of the metasurface‐CLC color filter is schematized on the CIE Chromaticity Diagram [37] in Figure 7d. Indeed, the plot of the colors corresponding to the different stages of the thermally induced phase transition is represented as circles (see zoomed view of Figure 7d). The yellowish green initial color (purple) is blue‐shifted to the green area (blue) and red‐shifted to the yellow area (red) to finally reduce its intensity (green). The comparison of this behavior with the bare CLC cell phase transition is highlighted in Figure S4. The plot of the CLC cell stages during the phase transition is represented in Figure S4a: The starting green color (purple) is first blue‐shifted to bluish green (blue), then red‐shifted to the deeper green (red), and it reduces its intensity at the isotropic state (green). Figure S4b shows the plot of the metasurface‐CLC phase transition on the entire CIE Chromaticity Diagram, showing the differences in the CLC behavior due to the structural diversity in the structural design. The presence of the AgNCs in the metasurface interferes with the CLC molecule's reorientation, slightly reducing their capacity to switch to different states. Consequently, the registered temperatures at the different stages also differ from those recorded for the bare CLC cell in Section 2.2: higher values are needed to start the phase transition.

This work addresses common limitations of traditional organic dyes by harnessing the thermoplasmonic effect of an engineered optical metasurface. The metasurface provides enhanced light–matter interaction at its plasmonic resonance, enabling highly localized and rapid temperature increases under moderate light intensity, as supported by prior studies [38]. Our approach allows control of the CLC pitch without affecting the CLC properties, and in addition, it introduces the novelty of multi‐optical components. Indeed, the reported concept exploits the integration of a CLC reflector (operating in the visible range) and an NIR absorber due to the metasurface component. Both optical components are actively controlled using the photo‐thermal properties of the metasurface. Conversely, even if adding a conventional dopant (e.g., organic dyes) could result in an active control of the CLC pitch because of the photo‐thermal properties of the dopant, it is worth noting that the dopants, e.g., organic dyes, suffer from photobleaching, thus producing long‐term instabilities. In addition, the same dopants could affect the CLC properties, lowering the transition temperature and producing sedimentation that will affect the CLC alignment.

Ultimately, the thermoplasmonic behavior of the optical metasurface allows for remote, light‐controlled tuning of the optical and morphological properties of the surrounding CLC material, opening new avenues for dynamic and reconfigurable photonic devices.

### 2.6 Reflection Dynamics Measurements

**FIGURE 8:**
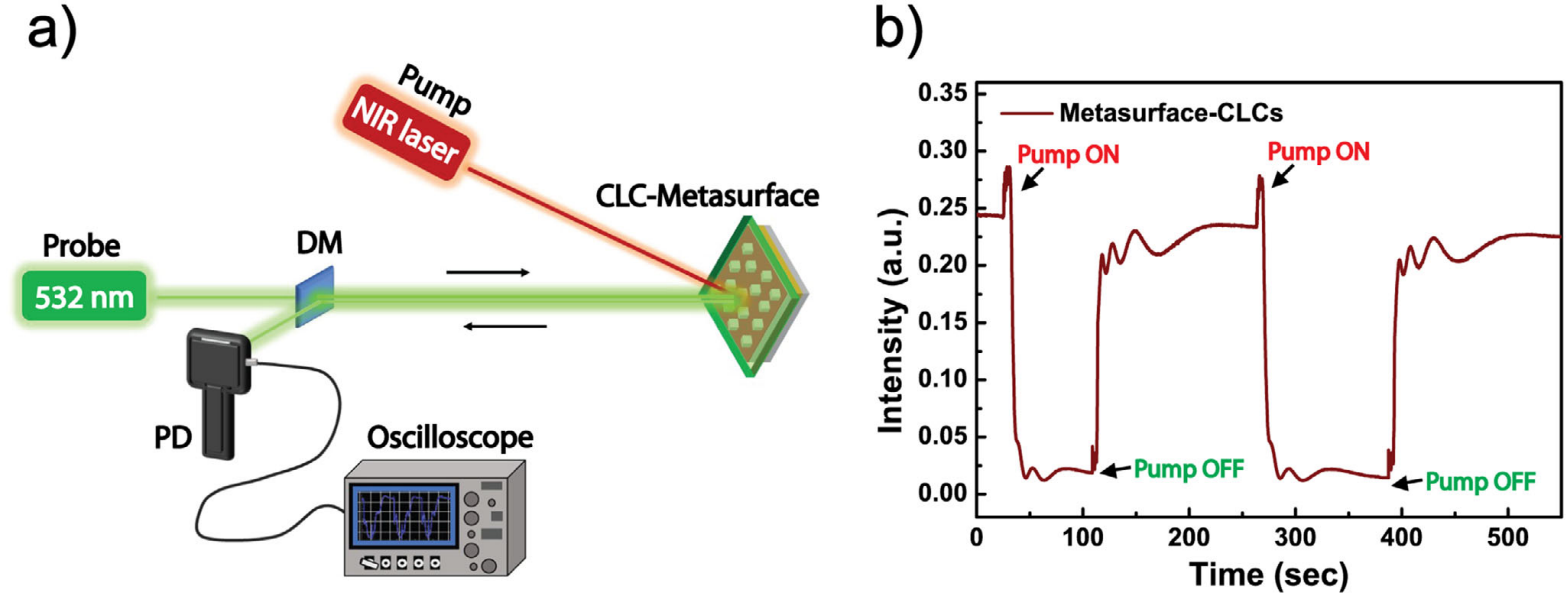
Reflection dynamics experiments. (a) Experimental setup for analyzing the reflection dynamics of the metasurface‐CLC sample. (b) Reflected intensity response of the metasurface‐CLC sample, triggered by switching the pump beam ON and OFF.

Reflection dynamics measurements were conducted to assess the reversibility and reproducibility of the spectral properties of the metasurface‐CLC samples under NIR laser activation. For this purpose, the optical setup shown in Figure 8a was implemented. The sample was probed with a 532 nm He‐Ne laser source, positioned within the center of the CLC reflection band, while the pump NIR laser beam (808 nm, 5.80 W/cm^2^) was alternately switched ON and OFF. It was observed that the reflected intensity changed from high to low values when the NIR beam was switched ON and vice versa when it was switched OFF (Figure 8b). This process revealed characteristic intensity oscillations associated with the photo‐thermal‐induced phase transition of the planarly aligned CLC film to the isotropic phase. The intensity decrease observed upon pump beam illumination aligns with the reduction of reflectivity at 532 nm for the metasurface‐CLC post‐irradiation (Figure 7b). The experiments on the metasurface‐CLC system were repeated multiple times to ensure consistency and reproducibility. This highlights the high reproducibility of the CLC metasurfaces, which outperform conventional dye‐based color filters in terms of thermal cycling stability and optical consistency. Additionally, the reflection dynamics demonstrate the system's rapid response, with an average response time of about 8 s. Moreover, this response time is significantly shorter than the 20 min irradiation required for metasurface‐CLC tuning in the main experiment (Section 2.4). The reflection dynamics experiment was conducted using two fully overlapping lasers (pump and probe beams), whereas the results reported in Section 2.4 (Figures 6, 7) were obtained by probing an area much larger than the pump beam. Specifically, the reflective probe's white‐light fiber covered an area three times larger than the pump beam. This setup enabled localized monitoring of the phase transition at the laser spot while capturing the averaged specular reflection spectra over the broader probed region. In the latter case, the homogeneous heating distribution induced by the pump beam required more time to fully equalize the temperature across the entire area probed by the white‐light beam. It is important to note that one effective way to improve the response times of the investigated metasurface‐CLC optical filter is to use an NIR pulsed laser. This type of laser can deliver the same amount of energy rapidly, eliminating the long illumination times associated with continuous wave lasers.

## 3 Conclusions

This study presented an innovative class of color filters combining a large‐area (1 cm^2^) NIR optical absorber (optical metasurface) with a visible Bragg mirror (CLC reflector) to create a nanostructured CLC‐based system. The component integrates a high‐birefringence, high‐transition‐temperature CLC with a broad Bragg reflection band (120 nm) and an optical metasurface optical absorber fabricated through a bottom‐up LbL self‐assembly technique. This design enables simultaneous NIR absorption and visible light reflection. The CLC film's reflection band can be dynamically tuned via the metasurface's photo‐thermal effect, achieving continuous control over an 80 nm range with a moderate irradiance of 5.80 W/cm^2^. The photo‐thermal induced transition in the CLC film also lowers its refractive index, actively modulating the optical metasurface's absorption band by approximately 10 nm. This multifunctional optical component is reversible and reproducible, as confirmed by reflection dynamics experiments. Ongoing work is focused on implementing polarization insensitivity, allowing the new components to function effectively under unpolarized light.

## 4 Experimental Section

### 4.1 Materials

Gold‐coated (Au) glass slides, 50 nm thick (1 cm × 1 cm), were purchased from AMSBIO. AgNCs (100 nm side, 1 mg) were purchased from Nanocomposix. The PEs poly(sodium 4‐styrenesulfonate) (PSS, Mw 70 000 Da) and poly‐(allylamine hydrochloride) (PAH, Mw 50 000 Da) were purchased from Merck. The high birefringence NLC 1825 was purchased from the Military University of Technology (Poland). Sodium chloride (NaCl) was purchased from Sigma–Aldrich. NOA 61 was purchased from Norlands. Planarly aligned glass substrates were purchased from the Military University of Technology (Poland). Millipore water was used in all of the procedures.

### 4.2 Fabrication of the Optical Metasurface

The optical metasurfaces were fabricated using an improved version of the protocol in ref. [23]. At first, the Au glass slides were washed with water and dried under a nitrogen flow. Subsequently, the PEM was constructed by immersing the slides for 5 min in PAH and PSS 1.6 mg mL^−1^ dissolved in 0.5 m NaCl in the sequence PAH‐PSS‐PAH‐PSS‐PAH and using 0.5 m NaCl for intermediate rinsing steps of 1 min. At the end, they were dried under a stream of nitrogen. AgNCs were finally immobilized through the last overnight immersion in the colloidal solution of AgNCs (OD 1.5) dispersed in MilliQ water. After the immersion, the substrates were washed with water and dried under a nitrogen stream to be characterized.

### 4.3 Preparation of Cholesteric Liquid Crystals

CLCs were prepared by adding 3 and 4 wt.% of S5011 left chiral dopant, purchased by Daken Chemical, to the NLCs 1825, achieving both CLCs materials with λ_B_ centered at 600 and 500 nm. The solutions were then mixed and maintained at 110°C on the hot plate to completely melt the chiral agent.

### 4.4 Photo‐Thermal Investigation of the Cholesteric Liquid Crystals

The metasurface‐CLC samples were analyzed by monitoring them when irradiated under a NIR laser, specifically a CW fiber‐coupled 808 nm laser source (MDL‐H‐808 from CNI Laser), which was the closest available wavelength to the absorption maximum among our laboratory equipment. Although there was a slight spectral offset, the metasurface's broad absorption profile ensures efficient photo‐thermal excitation at 808 nm. The temperature distribution and thermal profile on the metasurface‐CLC samples were recorded using a high‐resolution thermal camera (FLIR, A655sc), generating thermal images of 640 × 480 pixels with an accuracy of ± 0.2°C, ensuring accurate correlation between the optical response and the local temperature under NIR excitation. The software FLIR ResearchIR Max was used to record and process the thermal data acquired by the camera.

### 4.5 UV–Vis Reflectance Spectroscopy

The metasurface‐CLC samples were analyzed using reflectance spectroscopy. Measurements were conducted with an optical setup comprising a USB spectrophotometer (USB 2000 + XR1, Ocean Optics, FL, USA) equipped with a UV–vis light source (CLS 100; Leica, Vienna, Austria) connected to a reflective optical fiber. The samples were placed on a holder, and a CW fiber‐coupled 808 nm laser source (MDL‐H‐808 from CNI Laser) in the NIR range was integrated into the setup to control the sample's spectral response under laser illumination photo‐thermally. The setup geometry was designed to precisely probe the sample area, with the reflective fiber spectrophotometer positioned to overlap the laser‐activated region of the sample directly.

### 4.6 Hot Plate Investigation of Cholesteric Liquid Crystals

The thermal properties of the CLCs were initially investigated by heating them on a hot plate. A planarly aligned glass cell was infiltrated with CLCs, placed on the hot plate, and set to various temperatures ranging from 24°C to 130°C. During this process, optical spectra were collected by incorporating the hot plate into the reflective optical setup, while thermal images were captured using a FLIR A655sc thermal camera.

### 4.7 Reflection Dynamics Experiments

The system's optical setup, used to perform reflection dynamics of the metasurface‐CLC samples, consisted of a pump CW diode laser (Coherent Powerline) operating at 808 nm and a probe CW He‐Ne laser (Newport) operating at 532 nm. The transmitted intensity signal was recorded using a Thorlabs Si amplified photodetector PDA8A2, fixed gain, monitored using a Tektronix 3 Series MDO Mixed Domain Oscilloscope.

## Conflicts of Interest

The authors declare no conflicts of interest.

## Supporting information




**Supporting file 1**: marc202500339‐sup‐0001‐SuppMat.docx

**Supporting file 2**: marc202500339‐sup‐0002‐VideoS1.mp4

## Data Availability

The data that support the findings of this study are available from the corresponding author upon reasonable request.
