# Supplementary material for: Thermoplasmonic‐Controlled Optical Filters Based on the Combination of Chiral Liquid Crystals and Metasurfaces
Source: Macromol Rapid Commun. 2025 Jul 16;46(21):e00339. doi: 10.1002/marc.202500339 (PMC12590940; doi:10.1002/marc.202500339)
Supplement: Supplementary file 1 — Supporting file 1: marc202500339‐sup‐0001‐SuppMat.docx [file MARC-46-e00339-s002.docx]

Supporting Information

Thermoplasmonic-controlled Optical Filters based on the Combination of Chiral Liquid Crystals and Metasurfaces

Federica Zaccagnini*, Francesca Petronella, Michael E. McConney, Jonathan Slagle, Kwang-Un Jeong, Timothy J. Bunning, Luciano De Sio*

**SI 1. Hot plate heating of CLC cell**

The preliminary investigation of the CLC cell heated on a hot plate assessed the reversible blue-red shift of the CLC Bragg reflection band.


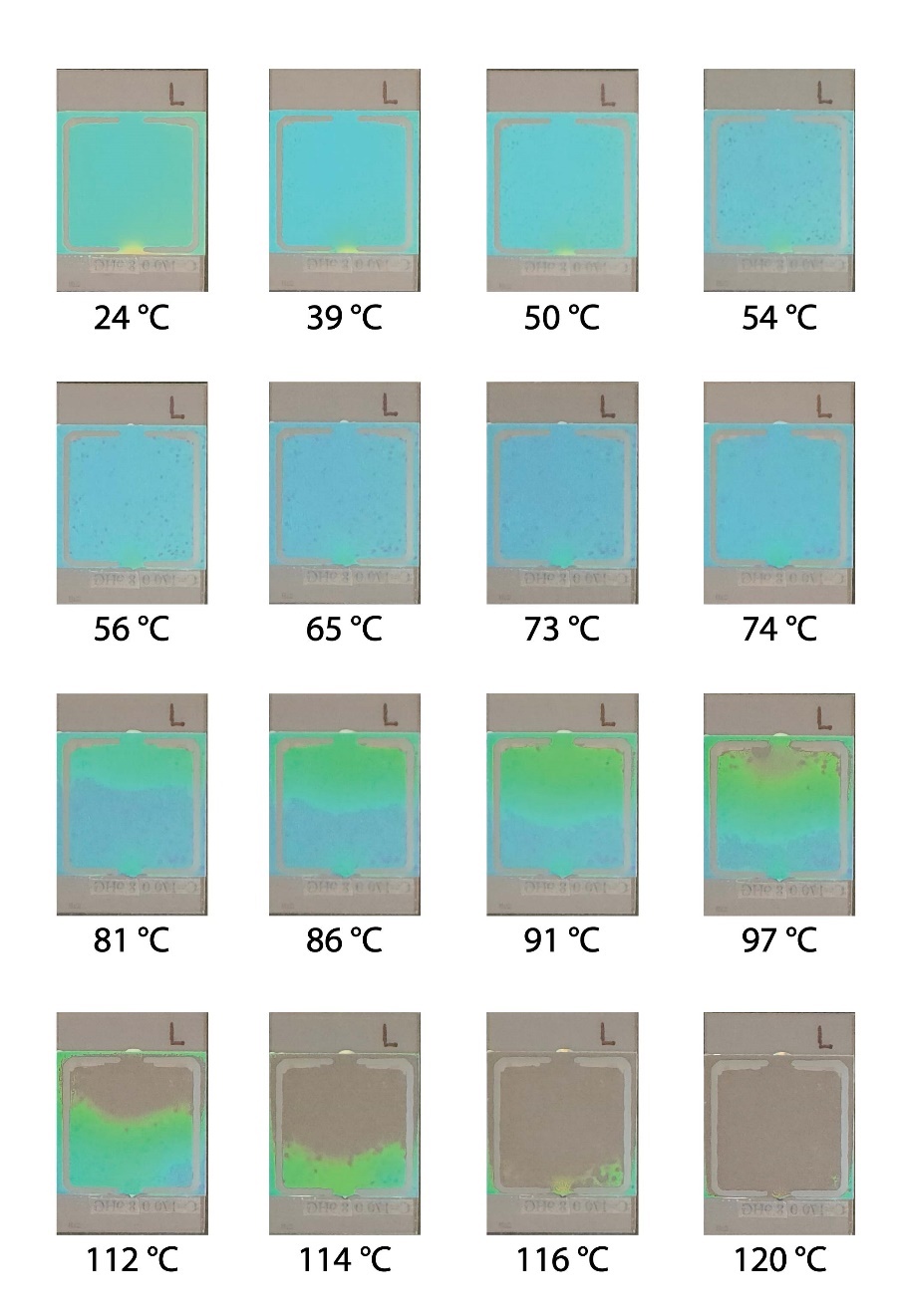


**Figure SI 1**. Gradual color change of the Left CLC cell when heated on a hot plate from 24 °C to 120 °C.

**Figure SI 1** highlights the color change for the different set temperatures. The green color visible at 24 °C suddenly changes into a dark green and a deep blue at 73 °C. This variation is ascribable to the blue shift of the Bragg reflection band. After that, as the set temperature increases, the deep blue is converted into green, reaching a lighter hue than the initial state at 97 °C. The second trend described is relatable to the subsequent red shift of the Bragg reflection band. For temperatures higher than 100 °C, the chiral-to-isotropic phase change gradually occurs. The CLC film goes entirely to the isotropic state at 120 °C.

**See Video SI 1**

**Video SI 1**. The 16x speed video of the heated Left CLC cell on a hot plate.

**Video SI 1** shows the CLC film's capability to recover the chiral state after the chiral-to-isotropic phase transition, proving the fast reversibility of the tunable color filter that withstands high temperatures without degrading its efficiency.

SI 2. Photo-thermal characterization of the random optical metasurface and of the CLC cell


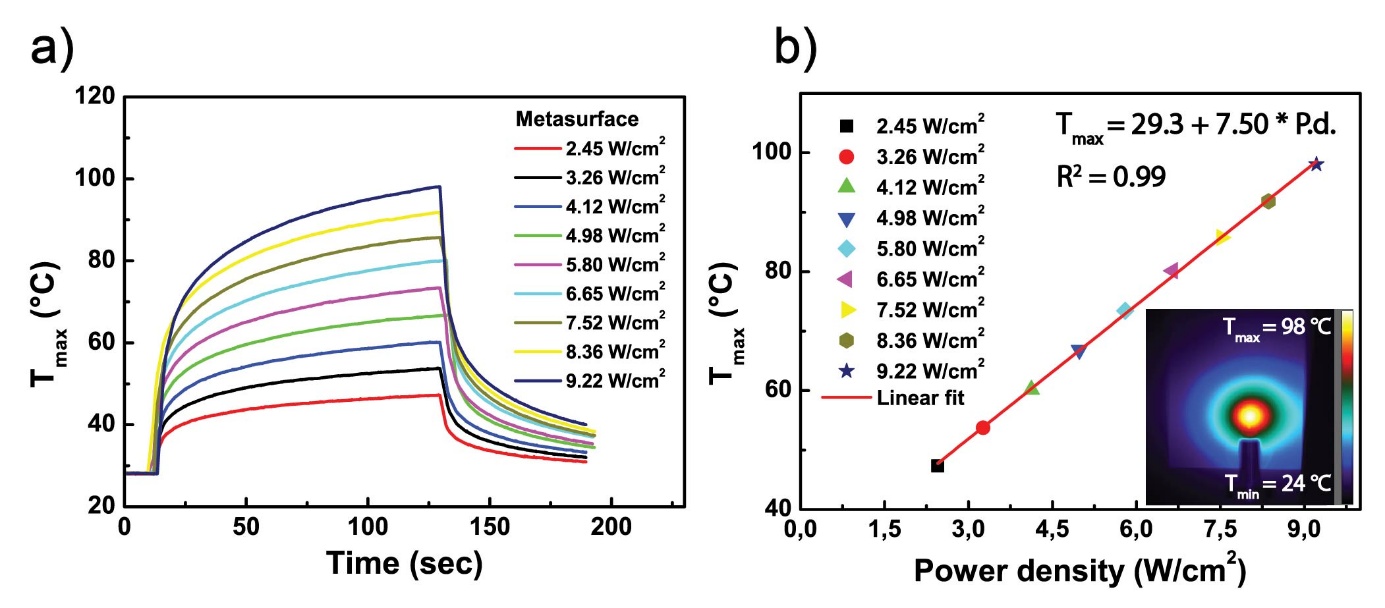


**Figure SI 2**. Photo-thermal characterization of the random metasurface. (a) Temperature increase observed upon irradiation of the optical metasurface with an 808 nm laser at varying power densities. (b) Maximum temperatures reached by the optical metasurface are a function of the experimental data points' NIR power density and linear fit (red line).

The same photo-thermal setup in **Figure 4a** was first used to monitor the photo-thermal properties of the bare metasurface, irradiating the sample with the NIR laser at different power density values. Maximum surface temperatures ranging from 47.2 °C to 98.0 °C were recorded for power densities between 2.45 W cm⁻² and 9.22 W cm⁻² (see **Figure SI 2a**). The thermographic image (inset of **Figure SI 2b**) highlights the peak temperature of 98.0 °C at 9.22 W cm⁻², while **Figure SI 2b** shows that the increase in temperature is approximately linear with power density. The realized bottom-up metasurface exceeds the photo-thermal response of the previous one realized in ref ^[1]^ of about 30°C since increasing the NCs density can enhance the absorption and thus the photo-thermal conversion of the metasurface at a specific wavelength. ^[2]^

Various research groups have explored the photo-thermal effects in plasmonic metasurfaces.^[3-8]^ As reported in **Table S11**, temperature values higher than 100 °C for moderate laser pump intensities or lower than 15 W/cm^2^ are not reported. In particular, under NIR irradiation, state-of-the-art metasurfaces reported a maximum temperature value of 50°C. Instead, our perfect absorber metasurface, due to its unique nature of perfect absorption behaviour, can reach temperatures of up to 120 °C. Indeed, under these experimental conditions, the CLC having a transition temperature close to 120 °C can achieve the largest shift of the reflection band.

**Table SI 1.** Summary table, comparing the maximum temperature reached by recently reported dielectric and plasmonic metasurfaces for a wide range of applications under an external stimulus.

| **Classification of the metasurface** | **Laser wavelength**  **(nm)** | **Laser intensity**  **(W/cm^2^)** | **Maximum temperature**  **(°C)** | **Presence of liquid crystal** | **Application field** | **Reference** |
| --- | --- | --- | --- | --- | --- | --- |
| Dielectric (TiO_2_-based) | n.a. | n.a. | 78.1°C | Yes (Cholesteric) | Holography | (3) |
| Dielectric (TiO_2_/Graphene)-based | 808 | 1.1 | 50 | no | Antibacterial, osteointegration | (4) |
| Metallic | 1030 | 0.4 | 45 | Yes (elastomers) | Beam stearing | (5) |
| Plasmonic | n.a. | n.a. | 35 | Yes (nematic) | Nanoprinting | (6) |
| Plasmonic | 532 | 14 | 80 | no | Antireflecting and antifogging | (7) |
| Plasmonic | 532 | n.a. | 70 | no | Energy harvesting | (8) |

**SI 3. Gamut color change on CIE Chromaticity Diagram**


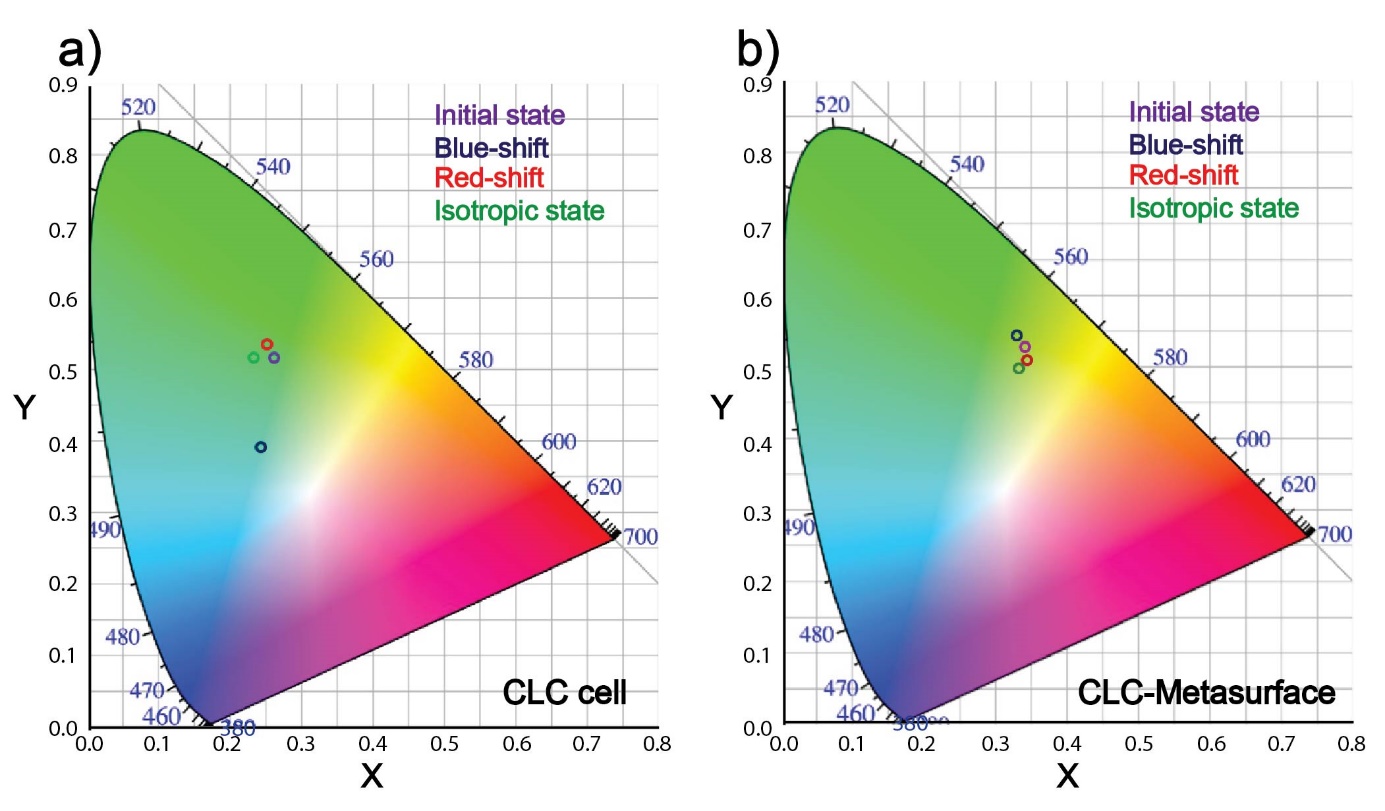


**Figure SI 4**. Gamut color change on CIE Chromaticity Diagram of a) the CLC cell under hot plate heating and of b) the metasurface-CLC under NIR irradiation.

The performance of the realized color filter is investigated plotting the intensities of the reflectance spectra acquired during the phase transition with the Matlab program CIE Coordinate Calculator for both the CLC cell and the metasurface-CLC. ^[9]^

References

[1] F. Petronella, T. Madeleine, V. De Mei, F. Zaccagnini, M. Striccoli, G. D'Alessandro, M. Rumi, J. Slagle, M. Kaczmarek, L. De Sio, *ACS Appl Mater Interfaces* **2023**.

[2] Ji-an Chen, Yuyuan Qin, Yubiao Niu, Peng Mao, Fengqi Song, Richard E. Palmer, Guanghou Wang, Shuang Zhang, and Min Han, *Nano Letters* **2023** *23* (15), 7236-7243

[3] J. Kim, J.-H. Im, S. So, Y. Choi, H. Kang, B. Lim, M. Lee, Y.-K. Kim, J. Rho, Dynamic Hyperspectral Holography Enabled by Inverse-Designed Metasurfaces with Oblique Helicoidal Cholesterics. Adv. Mater. **2024**, 36, 2311785.

[4] M. Yang, Y. Zhang, Z. Hou, J. Wu, F. Liu, J. Wu, K. W. Yeung, W. Qian, X. Liu, L. Kong, Y. Li, J. Qiu, G. Wang, NIR-I Light-Activated Antibiotic Delivery & PDT via TiO2/Graphene Metastructure for Enhanced Antibacterial Activity and Osseointegration of Ti Implants. Adv. Healthcare Mater. 2025, 14, 2500743.

[5] Zhuang, X., Zhang, W., Wang, K. et al. Active terahertz beam steering based on mechanical deformation of liquid crystal elastomer metasurface. Light Sci Appl **2023**, 12, 14.

[6] L. Zheng, Y. Liu, N. Zhang, X. She, C. Jin, Y. Shen, Liquid-Crystal-Powered Metasurfaces for Electrically and Thermally Switchable Photorealistic Nanoprinting and Optical Security Platform. Adv. Funct. Mater. 2025, 35, 2415104.

[7] F. Thorimbert, M. C. Rivadeneira, M. Faustini, Self-Assembling Cracks to Fabricate Antireflective Antifogging Metasurfaces. Adv. Optical Mater. 2025, 13, 2500079.

[8] Ji-an Chen, Yuyuan Qin, Yubiao Niu, Peng Mao, Fengqi Song, Richard E. Palmer, Guanghou Wang, Shuang Zhang, and Min Han. Nano Letters 2023 23 (15), 7236-7243

[9] Prashant Patil (2024). CIE Coordinate Calculator (https://www.mathworks.com/matlabcentral/fileexchange/29620-cie-coordinate-calculator), MATLAB Central File Exchange. December 29, 2024.]
